# A Calculation Model of the General Theory of Interaction Potentials for Stoichiometric Lanthanide Type Crystals: Applications to the *Cs*_2_*KLnCl*_6_ System

**DOI:** 10.1038/s41598-019-55695-6

**Published:** 2019-12-13

**Authors:** Andres Soto, Shanavas Shajahan, Roberto Acevedo

**Affiliations:** 1 Facultad de Ingeniería y Tecnología, Universidad San Sebastián, Bellavista 7, Santiago, 8420524 Chile; 2 Nano and Hybrid Materials Laboratory, Department of Physics, Periyar University, 636011 Salem, India

**Keywords:** Theory and computation, Condensed-matter physics

## Abstract

This article aims to develop a generalized model calculation model to be applicable to the general theory of interaction potentials with reference to the stoichiometric elpasolite type crystals. In this study, we have chosen to report both a theoretical model and a calculation strategy to undertake semi empirical calculations of thermodynamic properties, such as reticular energies and heats of formation for the series of systems such as: *Cs*_2_*KLnCl*_6_. We have also carried out quite a number of calculations for a variety of systems such as: *Cs*_2_*NaLnF*_6_*, Cs*_2_*NaLnCl*_6_*, Cs*_2_*NaLnBr*_6_*, Rb*_2_*NaLnF*_6_ *and Cs*_2_*KLnF*_6_ in the *Fm3m* space group since we aim to check the strengths and weaknesses of our model calculations. We have analyzed a substantial number of approximate theoretical models and have carried a formidable amount of computing simulations to estimate the reticular energies and the corresponding heat of formation for these type of crystal using a semi empirical model. We made use of the thermodynamic cycles of Born-Haber so as to get a broad view with reference to the accuracy of our semi empirical theoretical models. The problem itself is quite challenging since we have focused our attention upon trivalent lanthanide ions $L{n}^{+3}$ in the first inner transition series of the chemical elements: (*Ce, Pr, Nd, Pm, Sm, Eu, Gd, Tb, Dy, Ho, Er, Tm, Yb, Lu*). There are a significant amount of outstanding research works published in the literature with reference to structural analysis, one photon spectroscopy, vibrionic intensity model calculations and generalized models to deal with these kind of complex crystals. The calculated energy values associated with these observables seems to be most reasonable, and these follow the expected trends, as may be expected on both theoretical and experimental grounds. Both, the advantages and disadvantages of the current model calculations, have been tested against other previous calculations performed for this type of complex systems. It is of a paramount importance, the results obtained and reported in this article with regards to convergence tests as well as some master equations derived to account for the various contributions to the total energy. The Born-Mayer-Buckingham potential is carefully examined with reference to these lanthanide type crystals *Cs*_2_*KLnCl*_6_. Finally but not at last, the most likely sources for improvement are carefully discussed in this work. We strongly believe that there is enough room for improvement and have therefore initiated a new research program of activities tackling systems of well-known optical and structural properties.

## Introduction

For ionic crystals, a simple model suggests that the cohesive energy may be estimated adding all the contributions, derived from the long range coulombic interaction terms $(C)$, i.e. the Madelung energy. The potential function employed in these model is essentially electrostatic and depends mainly upon two conditions; the spatial geometrical surrounding of the electrostatic charges and also the relative distances between them. In addition, further refinements have been introduced over the last two decades, with reference to a number of more comprehensive and sophisticated physical models. In this current approach, the reticular energy is partitioned into three components; Coulombic (C), Born-Mayer (B-M) and Van der Waals (VDW). Utilizing a Born-Mayer-Buckingham type potential to estimate the three contributions, as mentioned above for the total energy. The parameters employed in these calculations were the value of “a” (lattice parameter of crystal) and the value of structural parameter “x” (the ratio of distance between lanthanide-halide pairs over lattice parameter). Our work represents a further step and it aims to estimate both, the reticular energies and the heats of formation, regarding the crystals such as $C{s}_{2}KLnC{l}_{6}$, in the $Fm3m$ space group. We have reported previous and preliminary calculations for systems such as: $C{s}_{2}NaLn{F}_{6},C{s}_{2}NaLnC{l}_{6},C{s}_{2}NaLnB{r}_{6},R{b}_{2}NaLn{F}_{6}$ and $C{s}_{2}KLn{F}_{6}$ in the $Fm3m$ space group with a fair degree of success^1,2^.

These systems have been the target of many experimental and spectroscopic studies^3,4^ and reference there in. We shall focus our attention upon intermolecular and intramolecular interaction terms, in the broad area of the thermodynamics, so the above-mentioned points will not be discussed here. In this article, we will concentrate on some physical aspects and convergence tests for the crystalline sums over the main polarization directions of the crystals. The origin and nature of these problems is indeed formidable, though and as we shall see the results are as good as could be expected and though, the lack of experimental data, these theoretical predictions follow the right trend and represents a challenge to develop some more elaborated and realistic models for these kind of crystals.

### The madelung constant and ewald method

#### Overture

As it was mentioned in the above section, the basic assumption of a simple model is to consider the potential associated with long range interactions, in an ionic crystal as essentially electrostatic in character. Thus, the potential energy function is currently written as follows:

1 $$V=\sum _{i < j}{V}_{i,j}=\sum _{i < j}\frac{{e}^{2}}{4\pi {\varepsilon }_{0}}\frac{{Z}_{i}{Z}_{j}}{{\rho }_{i,j}}$$

Where, ${\rho }_{i,j}$ is the relative distance among the $(i,j)$ charge-pair? The above identity may conveniently be expressed, say for a binary crystal $(AB)$ as given below:

2 $$V=\frac{{Z}_{+}{Z}_{-}{e}^{2}}{4\pi {\varepsilon }_{0}}\frac{1}{R}\sum _{i < j}\frac{1}{{r}_{i,j}}$$

In the above notation, $R$ is regarded as some kind of a reference distance, upon which the summation is run over, along the three polarizations directions of the crystal. This magnitude is such that ${r}_{i,j}$ becomes dimensionless (regarded as reduced coordinates for the crystal) and hence: ${\rho }_{i,j}=R\,{r}_{i,j}$. It is straightforward to realize that the term, say $\hat{A}=\sum _{i < j}\frac{\pm 1}{{r}_{i,j}}$ stands for result of the sums along the principal inertia axis of the crystal, when a given number of discrete charges are considered. It is rather straightforward to realize that the identity given by Eq. (2) should, in theory be both applicable and appropriate for crystals such as $AB(s)$.

When this Madelung constant $(\hat{A})$, is the purpose of a given calculation for a binary crystal, say $AB(s)$, the value used for $R$ is chosen so as to correspond to the smallest distance between the cation and the anion ions. There are also, other strategies which may be employed and these are based upon the choice of the lattice parameter as a unit of distance. The main goal of the current research work is to develop a general formalism both flexible and simple to undertake calculations with some utility and credibility for a variety of these and related crystals. The output of these calculations has to be independent of the model and the strategy followed throughout the course of the calculation.

From Eq. (1), we observe that $V=\sum _{i < j}{V}_{i,j}=\sum _{i < j}\frac{{e}^{2}}{4\pi {\varepsilon }_{0}}\frac{{Z}_{i}{Z}_{j}}{{\rho }_{i,j}}=-\frac{{e}^{2}}{4\pi {\varepsilon }_{0}}\frac{1}{R}\hat{A}\text{'}$, where $\hat{A}\text{'}=-\sum _{i < j}\frac{{Z}_{i}{Z}_{j}}{{r}_{i,j}}$. It is also reasonably to anticipate that these two identities are general so that they can be applicable to more sophisticated calculations for systems such as the lanthanide-type crystals.

#### Some additional comments about the Madelung constant

In a pioneer work, Madelung^5^ put forward a model to estimate the constant named after him, modeling the ionic crystal as periodic array of alternate charges in two and three dimensions covering the whole physical space. There have also been alternative schemes due to other workers to pursue the same objective^6–9^. Some of these research efforts are as follows:

#### Some further comments about the Ewald method

The determination of the total electrostatic energy for a crystal requires of a physical model and a mathematical procedure to be able to sum over the attractive and repulsive interaction terms between the ions. For a given crystal of n-atoms for each cell, there are k type of atoms so that $\mathop{\sum }\limits_{i=1}^{k}{n}_{i}=n$. In the current notation, ${n}_{i}$ represents the number of i- th type of atoms in a given cell carrying charges ${Z}_{i}$. There only one restriction which is related to the electro neutrality principle for the system, i.e. $\mathop{\sum }\limits_{i=1}^{k}{n}_{i}{Z}_{i}=0$. We shall denote by $R$, the smallest cation-anion distance and also $|{r}_{ij}|$ and $|{\overrightarrow{R}}_{k}+{\overrightarrow{r}}_{ij}|$, where ${\overrightarrow{R}}_{k}$ is employed to label the various cells in the crystal and ${\overrightarrow{r}}_{ij}$ corresponds to the relative distance between two ions in the same cell. We will also assume the existence of ${N}_{l}$ cells chosen as representative of the crystalline cell. When this procedure is followed, the Madelung constant may be expressed as given in Eq. (3):

3 $$\hat{A}\text{'}=-\frac{1}{2}\mathop{\sum }\limits_{i=1}^{n}[\mathop{\sum }\limits_{\begin{array}{c}j=1\\ j\ne i\end{array}}^{n}\frac{{Z}_{i}{Z}_{j}}{|{\overrightarrow{r}}_{ij}|}+\mathop{\sum }\limits_{k=1}^{{N}_{L}-1}[\mathop{\sum }\limits_{j=1}^{n}\frac{{Z}_{i}{Z}_{j}}{|{\overrightarrow{r}}_{ij}+{\overrightarrow{R}}_{k}|}]]$$

The above given summations (which involve alternate charges) show a complicated mathematical problem, since the convergence of these sums is rather slow and it does depend upon the procedure used. There are, though several models reported in the literature to tackle this kind of problems. We can mention, models as those reported by Ewald, P3M (particle-particle, particle-mesh) and FMM (fast multipole method)^10–13^.

In the current research work, the columbic contribution to the total energy was evaluated employing the method put forward by Ewald. A thorough analysis of the various calculation models and methodologies reported in the literature may be found in reference^13^. The method put forward by Ewald, modifies the procedure currently employs to estimate the sums of Eq. (3), by adding and subtracting two terms, so as to include within the whole a set of spherical symmetric charges softening using a charge density ${\hat{\rho }}_{i}(r)$, centered at the particle positions. The formulae employed by Ewald employs a Gaussian for the charge density, ${\hat{\rho }}_{i}(r)$. In practice, the sums involved in Eq. (3), may be partitioned into two terms; one of them defined in the reciprocal space (Fourier transform) and the other represented by a simple summation in the direct space (evaluated by means of the error function). Along these lines, the contribution corresponding to the reciprocal of the distance is written as given below^14^

4 $$\frac{1}{r}=\frac{2}{\sqrt{\pi }}{\int }_{0}^{\infty }{e}^{-{r}^{2}{t}^{2}}dt=\frac{2}{\sqrt{\pi }}[{\int }_{0}^{\alpha }{e}^{-{r}^{2}{t}^{2}}dt+{\int }_{\alpha }^{\infty }{e}^{-{r}^{2}{t}^{2}}dt]$$

The above expression introduces naturally the ideas about both the direct and the reciprocal spaces. Thus, the second term on the right hand side of the above identity may be expressed in a closed form^12^:

5 $$\frac{2}{\sqrt{\pi }}{\int }_{\alpha }^{\infty }{e}^{-{r}^{2}{t}^{2}}dt=\frac{erfc(\alpha r)}{r}$$

The strategy proposed to evaluate the reciprocal of the distance ($\frac{1}{r}$), then the first terms (right hand side) of Eq. (4) may be estimated using a Fourier transform, thus when the variable $s=-\frac{{K}^{2}}{4\,{t}^{2}}$, is introduced, then we write^14^

6 $$\frac{2}{\sqrt{\pi }}{\int }_{0}^{\alpha }{e}^{-{r}^{2}{t}^{2}}dt=\frac{1}{2{\pi }^{2}}{\int }_{-\infty }^{\infty }{d}^{3}K\frac{\exp (-\frac{{K}^{2}}{4{\alpha }^{2}})}{{K}^{2}}\exp (-i\overrightarrow{K}\cdot \overrightarrow{r})$$

When the above explained methodology and strategy is adopted, the Madelung constant $(A\text{'})$, may be expressed as a partition of two terms: (one in the reciprocal and the other in the direct spaces), as given below

7 $$A\text{'}=A{\text{'}}_{real}+A{\text{'}}_{reciprocal}$$

Next, the Cartesian coordinates for the n th-atom in the unit cell characterized by the unit vectors $({i}_{1},{i}_{2},{i}_{3})$ are introduced as: ${r}_{n,{i}_{1},{i}_{2},{i}_{3}}=({x}_{n,{i}_{1},{i}_{2},{i}_{3}},{y}_{n,{i}_{1},{i}_{2},{i}_{3}},{z}_{n,{i}_{1},{i}_{2},{i}_{3}})$.

The above may be defined as a function of the fractional coordinates of the unit cell $({u}_{n1},{u}_{n2},{u}_{n3})$ and the three translation vectors of the unit cell ${a}_{j}$, where:

8 $${a}_{j}=({a}_{jx},{a}_{jy},{a}_{jz}),\,j=1,2,3$$

$${r}_{n,{i}_{1},{i}_{2},{i}_{3}}=({u}_{1,n}+{i}_{1}){a}_{1}+({u}_{2,n}+{i}_{2}){a}_{2}+({u}_{3,n}+{i}_{3}){a}_{3}$$

Furthermore, the sum over the direct space $A{\text{'}}_{real}$ is currently written as^15^:

9 $$A{\text{'}}_{real}=\frac{1}{2}\mathop{\sum }\limits_{k=1}^{{n}_{\max }}{q}_{k}\mathop{\sum }\limits_{n=1}^{{n}_{\max }}{q}_{n}\mathop{\sum }\limits_{{i}_{1}={i}_{\min }}^{{i}_{\max }}\mathop{\sum }\limits_{{i}_{2}={i}_{\min }}^{{i}_{\max }}\mathop{\sum }\limits_{{i}_{3}={i}_{\min }}^{{i}_{\max }}\frac{erfc(\alpha |{r}_{k}-{r}_{n,{i}_{1},{i}_{2},{i}_{3}}|)}{|{r}_{k}-{r}_{n,{i}_{1},{i}_{2},{i}_{3}}|}$$

The above summation is run over all non negligible terms and does not include the interaction of the ion with itself in the same reference cell. The indexes *k* and *n* label the various ions in the unit cell, whereas the indexes ${i}_{1},\,{i}_{2}\,$ and ${i}_{3}$ are used to describe the periodic translations along the principal axes. Also, the Ewald sum in the reciprocal space is given by the identity^15^:

10 $$\begin{array}{c}A{\text{'}}_{reciproco}=\frac{1}{2}\mathop{\sum }\limits_{k=1}^{{n}_{\max }}\frac{-{q}_{k}}{\pi V}\mathop{\sum }\limits_{n=1}^{{n}_{\max }}{q}_{n}\mathop{\sum }\limits_{{m}_{1}={m}_{\min }}^{{m}_{\max }}\mathop{\sum }\limits_{{m}_{2}={m}_{\min }}^{{m}_{\max }}\mathop{\sum }\limits_{{m}_{3}={m}_{\min }}^{{m}_{\max }}\frac{Exp(\frac{-{\pi }^{2}{|{f}_{{m}_{1},{m}_{2},{m}_{3}}|}^{2}}{{\alpha }^{2}})}{{|{f}_{{m}_{1},{m}_{2},{m}_{3}}|}^{2}}\cdot \\ \,Cos[2\pi {f}_{{m}_{1},{m}_{2},{m}_{3}}\cdot ({r}_{k}-{r}_{n,0,0,0})]\,+\{\mathop{\sum }\limits_{l=1}^{{n}_{\max }}\frac{-\alpha {{q}_{l}}^{2}}{\sqrt{\pi }}\}\end{array}$$

In the above expression, the volume of the unit cell is given as $V={a}_{1}\cdot ({a}_{2}\times {a}_{3})$ and the vector denoted by ${f}_{{m}_{1},{m}_{2},{m}_{3}}$, may be expressed as given below:

11 $${f}_{{m}_{1},{m}_{2},{m}_{3}}=({m}_{1},{m}_{2},{m}_{3})\,[\begin{array}{ccc}{b}_{11} & {b}_{12} & {b}_{13}\\ {b}_{21} & {b}_{22} & {b}_{23}\\ {b}_{31} & {b}_{32} & {b}_{33}\end{array}]$$

Also, the three vectors in the reciprocal cell are given by the identities:

12 $${\overrightarrow{b}}_{j}\leftrightarrow (\begin{array}{c}{b}_{j1}\\ {b}_{j2}\\ {b}_{j3}\end{array})$$

where, $${\overrightarrow{b}}_{1}=\frac{{\overrightarrow{a}}_{2}\times {\overrightarrow{a}}_{3}}{V},\,{\overrightarrow{b}}_{2}=\frac{{\overrightarrow{a}}_{1}\times {\overrightarrow{a}}_{3}}{V}$$ y $${\overrightarrow{b}}_{3}=\frac{{\overrightarrow{a}}_{1}\times {\overrightarrow{a}}_{2}}{V}$$

### The Born – Mayer potential

#### The born repulsion potential

Within the assumptions and approximations of the rigid spheres model for a $MX$ type crystal, the ionic distance corresponding to the ${M}^{+}-{X}^{-}$ pair, may in a first approximation be estimated as a sum of ionic radius of the action and anion ions^16^.

Should the crystal be ionic, then there is no covalence. Also, it is well known that the atoms are not rigid spheres and that the equilibrium distance between them corresponds to an optimum equilibrium between the attractive and the repulsive forces at the global minimum of energy. Similarly, the interactions between ions decrease as the relative distance between they increase. From a physical viewpoint, the repulsive interaction may be rationalized in terms of the Pauli Principle, which indicates that the total wave function must be ant symmetric with respect to the interchange among electrons. This view indicates, that when a certain degree of overlapping occurs then the electrons behavior suggests that they should occupy upper energy levels and this turns out to increase the repulsion energy of the system. Born put forward whereby the repulsion force may be represented by the term $(\frac{B\text{'}}{{r}^{n}})$, where the parameters B’ and n are characteristic of the pair of ions in question. When a given crystal, it is only considered the columbic contributions to the energy as well as the term due to Born, then it is straightforward to write:

13 $$U=\frac{N{z}^{+}{z}^{-}A{e}^{2}}{4\pi {\varepsilon }_{0}r}+\frac{NB}{{r}^{n}}$$

The relationships between $B$ and $n$ is obtained, assuming that the crystal in equilibrium $(r={r}_{0})$, obeys the condition which indicates that the energy must be evaluated at the global minimum. While, searching the critical points, we obtain the identity:

14 $${(\frac{dU}{dr})}_{r={r}_{0}}=0=-\frac{N{z}^{+}{z}^{-}A{e}^{2}}{4\pi {\varepsilon }_{0}{r}_{0}^{2}}-\frac{nNB}{{r}_{0}^{n+1}}\,{\rm{and}}\,B=-\frac{A{z}^{+}{z}^{-}{e}^{2}}{n4\pi {\varepsilon }_{0}}{r}_{0}^{n-1}$$

And therefore, we obtain the identity:

15 $$U=\frac{N{z}^{+}{z}^{-}{e}^{2}A}{4\pi {\varepsilon }_{0}{r}_{0}}(1-\frac{1}{n})$$

The numerical value for “n” may be obtained from compressibility’s measurements in solids^16^.

#### The Born – Mayer Potential

When the potential function $U$ is plotted (logarithm scale) against the internuclear distance “r”, there are is a range of values, say ${r}_{\inf }\le r\le {r}_{\sup }$, within this, the graphic with reference to the function U(r) becomes linear and the potential may be written as follows^15,16^:

16 $${U}_{ij}(r)={A}_{ij}{e}^{-{b}_{ij}r}={A}_{ij}{e}^{-\frac{r}{{\rho }_{ij}}}$$

The above term is known as the Born – Mayer potential. The values for “${A}_{ij}$” and “${b}_{ij}$” remain constant for the atoms $(i,j)$ at the ${r}_{ij}$ distance, when the range used to estimated these parameters is obeyed.

Some researchers suggest that the parameters ${A}_{ij}$ and ${b}_{ij}$, listing their associated values in an individual form so as the ${A}_{i}$ and ${b}_{i}$ values are assumed to be representative of the i th-atom and so forth^17,18^. In this model, we employ the identities:

17 $$\begin{array}{c}{A}_{ij}=\sqrt{{A}_{i}{A}_{j}}\\ {b}_{ij}=\frac{{b}_{i}+{b}_{j}}{2}\end{array}$$

The calculations were performed for interactions between ions at distances smaller than $4\,\mathop{A}\limits^{0}$. Furthermore, it is interesting to observe that for some pairs (positive-positive charges); these interactions might be neglected, in particular for all those cases when the relative distance is bigger than this value. An exceptional situation occurs for interactions of the type ${F}^{-}-{F}^{-}\,,C{l}^{-}-C{l}^{-}$ and $B{r}^{-}-B{r}^{-}$ (first neighbors), and in all these cases and for first neighbors, these interactions are considered^3^.

Our knowledge and experience indicates that this kind of approximation is reasonable. This seems to be a consequence of the lanthanide type geometry.

### The Born – Mayer – Bückingham Potential. the effect of the Van der Waals potential

A further generalization of this potential may be achieved by summing up an additional contribution to the energy, the well known Van der Waals type potential.

18 $$U({r}_{ij})=\frac{{z}_{i}{z}_{j}{e}^{2}}{4\pi {\varepsilon }_{0}{r}_{ij}}+{A}_{ij}\exp [-\frac{{r}_{ij}}{{\rho }_{ij}}]-\frac{{C}_{ij}}{{r}_{ij}^{6}}$$

When this is done, then the model is known as the Born – Mayer – Buckingham. Potential^19,20^. The coefficients ${C}_{ij}$ are obtained from the model put forward by Slater - Kirkwood.

Next, we write:

19 $${C}_{ij}=\frac{3}{2}\sqrt{\frac{{e}^{2}{\hslash }^{2}}{{m}_{e}}}\frac{{\alpha }_{i}{\alpha }_{j}}{\sqrt{\frac{{\alpha }_{i}}{{N}_{i}}}+\sqrt{\frac{{\alpha }_{j}}{{N}_{j}}}}$$

In the above identity, “$\alpha$” stands for the mean value of the electric dipolar polarizability corresponding to the i th-atom, “${m}_{e}$” is the electron mass and “N” is related to the concept of an effective number of electrons involved^3,21–25^

**Figure 1:**
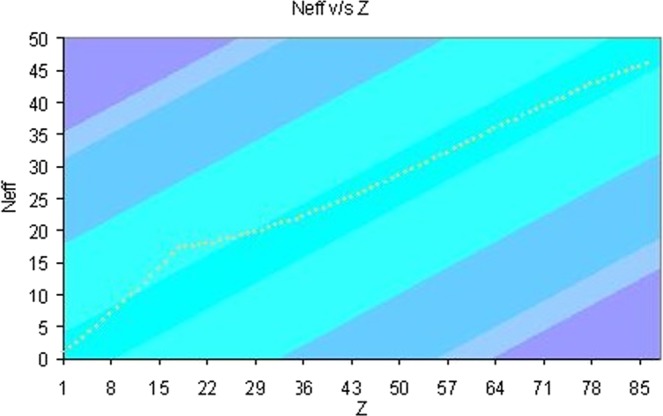
Dependence of these effective N-values with the atomic number.

The N-values for chemical elements up to 54 (Xe), may be obtained from the studies carried out by K. S. Pitzer^21,23^. This author worked with an effective N-value. The model put forward by Pitzer is a generalization to the scheme proposed by Slater-Kirkwood. For the sake of completeness, in Fig. 1, we display the dependence of these effective N-values with the atomic number (Z).

### Symmetry adapted model to estimate potentials

**Figure 2:**
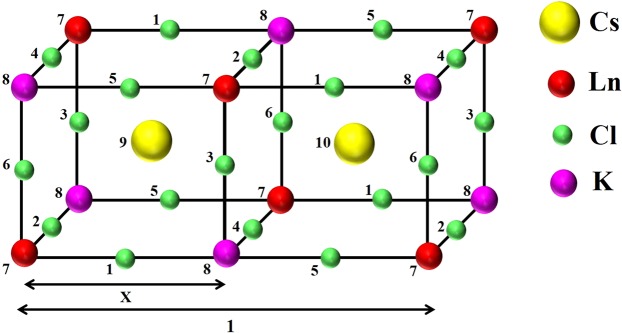
The stoichiometric lanthanide type crystal $C{s}_{2}KLnC{l}_{6}$.

For a ${M}_{2}NLn{X}_{6}$ stoichiometric lanthanide type crystal, it is trivial that the $M,N,Ln$ and $X$ atoms are not equivalent among themselves, though they are equivalent to any atom of the same species in the crystal. As we can see Fig. 2, illustrates the unit cell of some kind of stoichiometric elpasolite type crystal in the space group Fm3m and the “a” and “x” parameters are shown to illustrate the reader in a better way.

Thus, we may try to estimate the potential, employing the identity as given below:

20 $$\sum _{i < j}{U}_{ij}=\frac{(2\sum _{i}{U}_{M,i}+\sum _{i}{U}_{N,i}+\sum _{i}{U}_{Ln,i}+6\sum _{i}{U}_{X,i})}{2}\cdot {N}_{Avogadro}$$

In the above expression, the sum $\sum _{i}{U}_{atomo,i}$ runs over all the interactions of a given atom with all the others, excluding itself interactions. As for the Born-Mayer potential, the calculation is restricted to relative distances smaller than $4\mathop{A}\limits^{0}$ as it was mentioned earlier on in the text.

### The Born-Haber Thermodynamic cycle. Heats of formation

These cycles^3,26–28^ are currently employed to estimate the enthalpy values regarding the heats of formation for these lanthanide type crystals. For these purposes, several experimental data are required, such as the dissociation energies (D), the sublimation energies (S), electroafinity values (EA) and ionization potential (IP). Thus, it is straightforward for the variation of enthalpy, to write the identity^3^

21 $$\begin{array}{c}-\varDelta {H}_{form}=2S(M)+S(N)+S(Ln)+3D({X}_{2})+\\ \,2P{I}_{1}(M)+P{I}_{1}(N)+P{I}_{3}(Ln)-6E.A.(X)-{E}_{reticular}\end{array}$$

**Figure 3:**
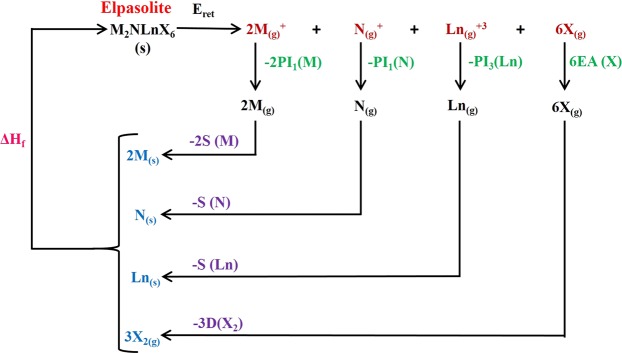
The Born-Haber Thermodynamic cycle for the stoichiometric lanthanide type crystals.

The Born-Haber thermodynamic cycle, may be illustrated by jeans of Fig. 3.

In the above Fig. 3, the reticular energy (${E}_{ret}$) may be assumed as the energy estimated by means of the Born-Mayer-Buckingham potential (the details of the calculations have been explained in the text, previously). All the experimental data involved in the proposed cycle, may be found in the literature. This allows us to make a reasonable and sound estimate of the heats of formation ($\Delta {H}_{form}$), for any stoichiometric lanthanide type crystals. We must emphasize that in a previous article^27^, we reported the data with reference to the Born-Mayer-Buckingham potentials and the heat of formation for a series of elpasolites type of crystals, such as: $C{s}_{2}KLn{F}_{6}$, $C{s}_{2}NaLn{F}_{6}$, $C{s}_{2}NaLnC{l}_{6}$, $C{s}_{2}NaLnB{r}_{6}$, $R{b}_{2}NaLn{F}_{6}$.

The next step is to complete these calculations reporting relevant data for systems such as $C{s}_{2}KLnC{l}_{6}$ in the Fm3m space group.

## Results and Discussion

Throughout the course of the current work, we have tackled different relevant issues, regarding the reticular energy (section-1), results of the calculations carried out by Marx and some aspects of the geometry of the crystals (section-2) and the Born-Haber thermodynamic cycles employed to estimate the heats of formation for these stoichiometric crystals (section-3). By means of this approach and the data reported in Appendix-1, it has been possible to advance forward and to estimate the reticular energies (as a sum of the coulombic, the Born-Mayer and the Van der Waals contributions) as well as the heats of formation ($\Delta {H}_{form}$) for the following family $C{s}_{2}KLnC{l}_{6}$,of lanthanide type crystals. The results obtained for the reticular energies and heats of formations are listed in Table 1, whereas in the Table 2, report the lattice parameters corresponding x-values.

**Table 1:** Energies for the series $C{s}_{2}KLnC{l}_{6}$. [KJ/mol].

Lanthanide	Born	V. Waals	Coulomb	E_reticular_	Δ*H*_*formation*_
Ce	950,025	−691,039	−6841,02	−6582,034	−2631,0
Pr	892,292	−703,324	−6879,29	−6690,322	−2704,3
Nd	929,965	−703,149	−6883,09	−6656,274	−2629,6
Pm	925,757	−709,6	−6904,27	−6688,113	−2599,9
Sm	985,609	−723,147	−6943,13	−6680,668	−2605,2
Eu	959,974	−737,643	−6982,65	−6760,319	−2550,6
Gd	943,096	−738,123	−6987,56	−6782,587	−2636,3
Tb	945,919	−746,545	−7011,29	−6811,916	−2633,6
Dy	1073,35	−762,714	−7052,45	−6741,814	−2554,2
Ho	951,164	−763,801	−7058,19	−6870,827	−2648,4
Er	954,522	−772,984	−7082,2	−6900,662	−2650,8
Tm	959,556	−782,597	−7106,67	−6929,711	−2655,0
Yb	1023,85	−801,048	−7150,02	−6927,218	−2582,2
Lu	1034,39	−803,126	−7156,97	−6925,706	−2612,9
Eu (*)	928,785	−725,344	−6952,34	−6748,899	−2539,1
Tb (*)	1048,47	−788,621	−7103,56	−6843,711	−2665,4

(*) Using data of Appendix-1.3–1.5 and compared with Table 3 for coulombic interaction.

**Table 2:** The lattice parameters “a” and the free structural parameter “x” for the series $C{s}_{2}KLnC{l}_{6}$.

Lanthanide (Ln)	a [pm]	X
Ce	1124,37	0,235
Pr	1123,01	0,233
Nd	1121,52	0,234
Pm	1119,90	0,233
Sm	1118,16	0,232
Eu	1116,30	0,230
Gd	1114,31	0,231
Tb	1112,20	0,230
Dy	1109,96	0,229
Ho	1107,60	0,229
Er	1105,12	0,229
Tm	1102,51	0,228
Yb	1099,77	0,227
Lu	1096,91	0,228
Eu (*)	1116,30	0,232
Tb (*)	1112,20	0,226

(*) data corresponding to experimental values reported in Table 3, Appendix-1.3–1.5.

V.Marx et al^3,26,29^. focused their attention upon systems such as K_2_NaScF_6_, K_2_NaGaF_6_, Cs_2_LiYCl_6_, CsLiLuCl_6_, Cs_2_NaBiCl_6_, Cs_2_NaYCl_6_, Cs_2_NaYBr_6_, Cs_2_NaHoBr_6_, Cs_2_KScCl_6_, Cs_2_KEuCl_6_, Cs_2_KTbCl_6_. It is straight to appreciate, that only for crystals such as: CsLiLuCl_6_, Cs_2_NaHoBr_6_, Cs_2_KEuCl_6_ and Cs_2_KTbCl_6_ the calculations reported by these authors included the coulombic energies, see Table 3. For illustrative purposes, we report for the above two crystals, $C{s}_{2}KEuC{l}_{6}$ and $C{s}_{2}KTbC{l}_{6}$, the coulombic contribution so as to compare the values reported by Marx *et al*.^26^ and the calculated in the current work. The units are in KJ/mol. (the values reported in the current work are given in square brackets).

**Table 3:** Coulombic energies. The “a” y “x” values taken from^24^.

Crystal	a [pm]	x	E_Coulombic_ [kJ/mol]
$C{s}_{2}KEuC{l}_{6}$	1116.3(3)	0.2317(8)	− 6966
$C{s}_{2}KTbC{l}_{6}$	1112.2(3)	0.2257(11)	− 7119

The above data should be compared with those (*), display in Table 1. We observe values very close among themselves. The difference are about 16 [KJ/mol] and the error is smaller thatn 0,2%. The conclussions with reference to the Born and Van der Waals model calulations have been reported in^30^ and will not be repeated here. With reference to the heats of formation for these crystals, we have re calculated these values using our strategy and model calculation since the differences are mainly shown from the calculation of the Van der Waals energies, though the lack of experimental data for the various families of lanthanide type crystals studied in this work, we have evidence that the calculations reported in the actual work for $C{s}_{2}KLnC{l}_{6}$, follow the expected trend and have the right order of magnitudes^29,31–33^. The thermodynamic cycles of the Born-Haber types have introduced in this article and the calculated values tabulated. Finally, the data employed throughout the course of the current work is given in Appendix-1, where the data given is as follows: 1.1: ionic radius for the lanthanides, 1.2: functions to estimate the lattice parameters as a function of the ionic radius, 1.3: experimental data for the lattice parameters in stoichiometric lanthanide crystals, 1.4:calculated lattice parameters(using the fittings given in 1.2), 1.5: x-parameter crystals, 1.6:electric polarizabilities for a series of ions, 1.7: Born-Mayer parameters, 1.8:Born-Mayer parameters for the pairs (lanthanide-halide), 1.9:ionization potentials for the trivalent lanthanides and 1.10:ionization potentials for the alkaline metals, 1.11:electroafinities for the halide ions, 1.12:bonding energies, 1.13: sublimation heats for metal ions, 1.14: estimated values for ${N}_{eff}$ and 1.15: estimate of the Born-Mayer for the lanthanide-halide pairs^34–39^.

## Conclusions

This research work aims to advance our understanding of the structural and energetic magnitudes which controls some relevant aspects of the thermodynamics for a series of five chosen lanthanide type crystals. We have focused our attention upon various model calculations, reported in the literature to model interaction potentials and to also estimate relevant energetic magnitudes. The approach was pioneered reported by Born-Mayer-Buckingham (B-M-B) and the model assumes appropriate to estimate the overall energy as a sum of pair energies. Nevertheless, the cohesive energy may be defined as the energy needed to obtain the crystal from their associated ions in the gas phase. As we mentioned in the text, within the framework of this model, the total energy may be understood as a sum of three contributions, though we are aware that for the stoichiometric lanthanide crystals a better description for the potential may be achieved by introducing the contribution to the total potential due to the polarization of the halide ions.

## Supplementary information

A Calculation Model of the General Theory of Interaction Potentials for Stoichiometric Lanthanide Type Crystals: Applications to the Cs2KLnCl6 System
