# Supplementary material for: A Calculation Model of the General Theory of Interaction Potentials for Stoichiometric Lanthanide Type Crystals: Applications to the Cs2KLnCl6 System
Source: Sci Rep. 2019 Dec 13;9:19088. doi: 10.1038/s41598-019-55695-6 (PMC6911071; doi:10.1038/s41598-019-55695-6)
Supplement: Supplementary file 1 — A Calculation Model of the General Theory of Interaction Potentials for Stoichiometric Lanthanide Type Crystals: Applications to the Cs2KLnCl6 System [file 41598_2019_55695_MOESM1_ESM.pdf]

A Calculation Model of the General Theory of Interaction Potentials for Stoichiometric Lanthanide Type Crystals: Applications to the Cs_2KLnCl_6 System

Andres Soto[a], Shanavas Shajahan[b], Roberto Acevedo[a*]

[a]*Facultad de Ingeniería y Tecnología. Universidad San Sebastián, Bellavista 7, Santiago 8420524, Chile.*

[b]*Nano and Hybrid Material Laboratory. Department of Physics. Periyar University Salem 636011. India*

**Corresponding author.*

Email address: roberto.acevedo.llanos@gmail.com (Roberto Acevedo)

Supplementary Section

APPENDIX-1

Appendix-1.1 – Ionic radius for the lanthanide ions[30]

Atom	Ionic radius [pm]
**Ce**	101
**Pr**	99
**Nd**	98
**Pm**	97
**Sm**	96
**Eu**	95
**Gd**	94
**Tb**	92
**Dy**	91
**Ho**	90
**Er**	89
**Tm**	88
**Yb**	87
**Lu**	86

Appendix-1.2 – Functions to estimate the lattice parameter (a) as a function of the ionic radius (r), for some lanthanide type crystals.

Cs_2KLnCl_6 :

$$a = 990.399 + 5{,}92229 \cdot r - 0.0622835 \cdot r^2$$

(*) obtained by fitting the data given in Appendix-1.3.

Appendix-1.3 – Experimental data for the lattice parameters series Cs_2KLnCl_6 [27].

Lanthanide	Sc	Ce	Tb
**Lattice parameter [pm]**	1087,3	1116,3	1112,2

Appendix-1.4. – Calculated lattice parameters [pm], using the data from Appendix- 1.2.

**Lanthanide**	Cs_2KLnCl_6
**La**	1124,37
**Ce**	1123,01
**Pr**	1121,52
**Nd**	1119,90
**Pm**	1118,16
**Sm**	1116,30
**Eu**	1114,31
**Gd**	1112,20
**Tb**	1109,96
**Dy**	1107,60
**Ho**	1105,12
**Er**	1102,51
**Tm**	1099,77
**Yb**	1096,91
**Lu**	1124,37

Appendix -1.5 – x-parameters for the crystals.

Let x be:

$$x = \frac{dis\tan ce(lanthanide - halide)}{Lattice\, parameter}$$

Where, we have employed the expression due to Shoemaker- Stevenson tipe:

$dist_{Ln-ha\log} = R_{Ln} + R_{halide} - 0{,}095 \cdot \left|\chi_{Ln} - \chi_{halide}\right|$; where R_{Ln} , R_{halide} are the ionic radius and χ_{Ln} , χ_{halide} represent the corresponding electronegativy values in the L.Pauling scale.

The corresponding x-values are tabulated as given below:

The x-values for some selected lanthanide type crystals

**Lanthanide**	Cs_2KLnCl_6
**Ce**	0,235
**Pr**	0,233
**Nd**	0,234
**Pm**	0,233
**Sm**	0,232
**Eu**	0,230

**Gd**	0,231
**Tb**	0,230
**Dy**	0,229
**Ho**	0,229
**Er**	0,229
**Tm**	0,228
**Yb**	0,227
**Lu**	0,228

Appendix-1.6 – Mean electric polarizabilities [A^3]

Ion	Polarizabilities [A^3]
**Cs^+**	2,420
**K^+**	0,830
**Cl^-**	3,660
**Ce^{+3}**	2,147
**Pr^{+3}**	2,110
**Nd^{+3}**	2,073
**Pm^{+3}**	2,036
**Sm^{+3}**	2,000
**Eu^{+3}**	1,964
**Gd^{+3}**	1,928
**Tb^{+3}**	1,893
**Dy^{+3}**	1,859
**Ho^{+3}**	1,825
**Er^{+3}**	1,791
**Tm^{+3}**	1,757
**Yb^{+3}**	1,724
**Lu^{+3}**	1,692

Data from Kittel **[33]** and Tosi **[34]**

Appendix-1.7 – Born-Mayer parameters [35-37]

Ion pairs	A [ev]	ρ [A]
**Cl^- - Cl^-**	1187,758	0,3399
**Cl^- - Cs^+**	4904,516	0,3232
**K^+ - Cl^-**	4117,9	0,3048

Appendix-1.8 – Born-Mayer parameters for the pairs lanthanide-halide ions

Pair ions (*)	A [ev]	ρ [A]
$La^{+3} - Cl^{-}$	3276,35	0,3349
$Ce^{+3} - Cl^{-}$	3390,35	0,3349
$Pr^{+3} - Cl^{-}$	2958,11	0,3349
$Nd^{+3} - Cl^{-}$	3099,00	0,3349
$Pm^{+3} - Cl^{-}$	2985,35	0,3349
$Sm^{+3} - Cl^{-}$	1965,57	0,3552
$Eu^{+3} - Cl^{-}$	2792,35	0,3349
$Gd^{+3} - Cl^{-}$	2706,35	0,3349
$Tb^{+3} - Cl^{-}$	2623,35	0,3349
$Dy^{+3} - Cl^{-}$	1117,00	0,3832
$Ho^{+3} - Cl^{-}$	2465,35	0,3349
$Er^{+3} - Cl^{-}$	2390,35	0,3349
$Tm^{+3} - Cl^{-}$	2321,35	0,3349
$Yb^{+3} - Cl^{-}$	1670,00	0,3515
$Lu^{+3} - Cl^{-}$	1450,23	0,3587

(*) MEG Valerio. Private communication.

Appendix-1.9 – Ionization potentials for the lanthanide ions [38]

$$M_{(g)} \rightarrow M^{+3}{}_{(g)} + 3e, \quad E_{tot}$$

Lanthanide	1er pot. [ev/át]	2do pot. [ev/át]	3er pot. [ev/át]	E_{tot} [ev/át]	E_{tot} [KJ/mol]
Ce	5,539	10,850	20,198	36,587	3530,0
Pr	5,470	10,550	21,624	37,644	3632,0
Nd	5,525	10,730	22,100	38,355	3700,6
Pm	5,582	10,900	22,300	38,782	3741,8
Sm	5,644	11,070	23,400	40,114	3870,3
Eu	5,670	11,241	24,920	41,831	4036,0
Gd	6,150	12,090	20,630	38,870	3750,3
Tb	5,864	11,520	21,910	39,294	3791,2
Dy	5,939	11,670	22,800	40,409	3898,8
Ho	6,022	11,800	22,840	40,662	3923,2
Er	6,108	11,930	22,740	40,778	3934,4
Tm	6,184	12,050	23,680	41,914	4044,0
Yb	6,254	12,170	25,050	43,474	4194,5
Lu	5,426	13,900	20,959	40,285	3886,8

Appendix-1.10 – Ionization potential for the Alkaline atoms[38]

$$M_{(g)} \to M^{+}_{(g)} + 1e, \quad En1$$

Metal	En1 [ev/col]	En1 [KJ/col]
**K**	4,34	418,7
**Cs**	3,89	375,3

Appendix-1.11 – Electroafinity values for the Halide atoms[39]

Halide	E.A. [ev/mol]	E.A. [KJ/mol]
**Cl**	3,7	358,0

Appendix-1.12 – Bonding energies [39]

Structure	D [Kcal/mol]	D [KJ/mol]
Cl_2	57,8	242,0

Appendix-1.13 – Sublimation energies [39, 40]

Metal	S [KJ/mol]	Metal	S [KJ/mol]
**K**	90,9	**Gd**	397,5
**Cs**	80,0	**Tb**	388,7
**Ce**	422,6	**Dy**	290,4
**Pr**	355,6	**Ho**	300,8
**Nd**	327,6	**Er**	317,1
**Pm**	348,0	**Tm**	232,2
**Sm**	206,7	**Yb**	152,1
**Eu**	175,3	**Lu**	427,6

Appendix-1.14 – The N_{eff} -values.

The values for N_{eff} were obtained by fitting those suggested by Pitzer **[22]**. In this article, we have estimated the values for this scalar for noble gases, taking the arithmetic average corresponding to the sum of all electrons and the located at the outer shell. (This approximation may be applicable to Rn (Z=86) and all the others have been tabulated by Pitzer.

Noble gas	N eff	N outer shell	Z	N eff (estimated)
**He**	**1,7**	2	2	2
**Ne**	**8,8**	6	10	8
**Ar**	**17,3**	6	18	12
**Kr**	**22,2**	6	36	21
**Xe**	**30,6**	6	54	30
**Rn**	-	6	86	**46**

The numbers in black bolds allow us to obtain these values for the other atoms. In the article by Scott and Scherega, the N_{eff} numbers were plotted against the atomic number, up to Xe **[24]**. In recent calculations, we have obtained the following correlations, as listed below:

$$f[Z] = 0{,}14375 + 0{,}75625 \cdot Z + 0{,}0109375 \cdot Z^2$$

$$g[Z] = 18{,}52932353 - 0{,}28724591 \cdot Z + 0{,}01351643 \cdot Z^2 - 0{,}00007514 \cdot Z^3$$

$$N_{eff}[Z] = \begin{cases} f[Z] & 1 \le Z \le 18 \\ g[Z] & 18 < Z \le 86 \end{cases}$$

The function for $N_{eff}[Z]$, reproduce the values reported by Pitzer and also for any atom.

Appendix-1.15 – The Born-Mayer parameters for the pairs lanthanide-halide ions

The calculation of the Born-Mayer parameters corresponding to a pair; a trivalent lanthanide-halide ions may be achieved according to the following strategy.

We need to write the expression:

$$U_{ij}(r) = A_{ij} e^{-b_{ij} r} = A_{ij} e^{-\frac{r}{\rho_{ij}}}$$

Where, we identify the A_{ij} and the ρ_{ij} parameters as a function of some additional terms as shown below **[34]**:

$$A_{ij} = f(\lambda_i + \lambda_j) Exp\left[\frac{(R_i + R_j)}{(\lambda_i + \lambda_j)}\right]$$

$$\rho_{ij} = \frac{1}{b_{ij}} = (\lambda_i + \lambda_j)$$

Here λ_k is the well-known hardness parameter, R_k is the ionic radius corresponding to the kth atom. For illustrative and computational purposes, we employed the values reported by Tatiplinar

et al. **[40].** These estimate values were calculated for systems such as LnX_3, where Ln stands for a trivalent lanthanide ion and X for a halide ions. Furthermore, $f = 0.05\left[e^2 / \overset{0}{A} \right]$ for the Chloride and Bromide ions **[34]**.
